# Position-specific workload of professional rugby union players during tactical periodization training

**DOI:** 10.1371/journal.pone.0288345

**Published:** 2024-03-29

**Authors:** Xiaopan Hu, Simon Boisbluche, Kilian Philippe, Olivier Maurelli, Xiangyu Ren, Shichang Li, Bo Xu, Jacques Prioux

**Affiliations:** 1 Sino-French Joint Research Center of Sport Science, College of Physical Education and Health, East China Normal University, Shanghai, China; 2 Movement, Sport, and Health Sciences Laboratory, Rennes 2 University, Bruz, France; 3 Department of Sport Sciences and Physical Education, École Normale Supérieure de Rennes, Bruz, France; 4 Rugby Club Vannes, French Rugby Federation, Vannes, France; 5 Movement, Balance, Performance, and Health Laboratory, University of Pau and Pays de l’Adour, Tarbes, France; 6 Muscle Dynamics and Metabolism Laboratory, University of Montpellier, Montpellier, France; Mugla Sitki Kocman University: Mugla Sitki Kocman Universitesi, TURKEY

## Abstract

The positional workload characteristics in rugby union on three acquisition days (i.e. strength, endurance, and speed days) of tactical periodization are still relatively unknown. Therefore, the primary aim of this study was to shed light on the positional external workload variables (10 Hz Global Positioning System and accelerometer microtechnology) and internal workload indicators (the session rating of perceived exertion) of players in a professional rugby union team by utilizing and comparing two tactical periodization models. Twenty-six male players (15 forwards and 11 backs) were recruited from a French second-division rugby club. Data were obtained over 10 weeks of in-season home games: a total of 780 observations were analyzed. Student’s *t*-test observed different external workload profiles between positions among acquisition days. Mean external workload values, except PlayerLoad_slow_, were significantly higher (*p*≤0.01; effect size: 0.41–1.93) for backs than forwards for all acquisition days. Moreover, forwards perceived a higher internal workload than backs on the strength day of both models. The findings demonstrate that applying these two tactical periodization models could result in effective rugby union training. Validating external and internal workload characteristics on tactical periodization acquisition days enables extensive analysis of training load monitoring data; these data can be utilized to discover the unique characteristics of each position and design position-specific acquisition days to improve performance.

## Introduction

From academy systems to elite sports, coaches have applied periodization models for many decades, indicating widespread cultural acceptance [1, 2]. Periodization is an essential aspect of the training structure and player development in team sports [1]. The recently developed tactical periodization model offers a holistic training concept that targets all training factors (e.g., physical, psychological, technical, and tactical) under the “supra-dimension” of tactical training [2]. In tactical periodization, the weekly pattern between two consecutive games is referred to as the “morphocycle” [3]. In a single-game morphocycle, there are three main acquisition days: strength, endurance, and speed days, focusing on maintaining or developing the respective physical capacities [4, 5]. From a biological perspective and based on methodological principles of tactical periodization, no two acquisition days within the morphocycle repeat the same physical stimulus in order to provide recovery time for distinct physical qualities [3, 4, 6].

Tactical periodization has presented sound alternatives to traditional periodization in team sports, including rugby union [2, 7]. Rugby is a dynamic and complex intermittent contact sport with position-specific demands. The position groups in rugby union can be divided into eight forwards and seven backs; forwards are involved in more collisions and backs cover more high-intensity running distances [8, 9]. Typically, forwards engage in 0.51 collisions per minute on a single competition [10]. These movements mainly occur during the tackle, ruck, maul, and scrum events and comprise 14% of the total match time [10–12]. Conversely, the role of backs is much more focusing on utilizing the space to run at high-velocity zones [10, 12]. Therefore, locomotor analyses reveal that backs experience fewer collision (0.27 collisions per minute), comprising only 2% of game time [10, 11]. Given the contrasting match demands between forwards and backs, training models should be chosen according to the needs of players’ positions [9].

Coaches and sports scientists use various methods, especially workload monitoring, to optimize specific training programs [8]. Workload can be categorized as measurements of external or internal workload, based respectively on the work performed by players (measured using e.g., the global positioning system [GPS]) or their subjective physiological and psychological response (e.g., the rating of perceived exertion [RPE]) [8, 9]. Technological and analytical advancements have created new opportunities to quantify workload in greater detail than ever before. However, despite the existence of numerous workload monitoring studies on rugby, no researchers have investigated the workload characteristics of rugby union players according to difference positions or distinct tactical periodization approaches. Thus, little is known about the workload characteristics and positional responses when applying tactical periodization in rugby union [2].

Responding to this research gap, this study aimed to be the first to describe the workload characteristics of a professional rugby union team between position units across three acquisition days utilizing two tactical periodization models (Model I and Model II). We first hypothesized that there would be significant workload variations across acquisition days and models for each position. Second, we hypothesized that substantial differences would exist between forwards and backs during the tactical periodization training period. These findings will provide valuable insights into tactical periodization and may be applied to rugby union in-season training. Specifically, a comparison of the positional units’ workload profiles on different acquisition days could assist team coaching staff in better understanding the training responses of tactical periodization to improve the intervention plans.

## Materials and methods

### Experimental approach to the problem

**Fig 1:**
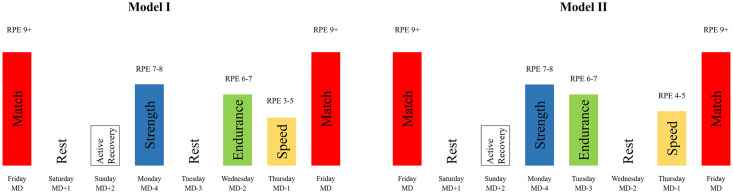
The two tactical periodization morphocycle models used for the practice session of a professional rugby union team. The height of each column represents the estimated load in arbitrary units. Abbreviations: RPE = rating of perceived exertion; MD = match day. Reprinted from [Xiaopan Hu, Rugby Club Vannes] under a CC BY license, with permission from [Rugby Club Vannes], original copyright [2020–2021].

In this study, we measured external and internal workload of different positions under tactical periodization training during the professional French National Rugby League (LNR) games weeks across the 2020 and 2021 seasons. In the 10-week experiment period, the external workload was monitored on each acquisition day using GPS technology, and the session RPE (s-RPE) was used at every on-field session to quantify the internal workload. We designed two morphocycles (Model I and Model II) based on the concept and principles of tactical periodization (Fig 1) [4, 7, 13]; each model contained a strength day (StD, Monday in both models), endurance day (EnD, Wednesday in Model I, Tuesday in Model II), and speed day (SpD, Thursday in both models).

### Subjects

The study’s inclusion criteria were (a) all data were collected during home game weeks to avoid the influence of different training conditions and travel associated with away games [14], and (b) only home game weeks where the tactical periodization morphocycle training model was used were included. The study’s exclusion criteria were (a) data from the first usage of the tactical periodization models in a game week, even of it was a home game, and (b) data relating to players who were injured, resulting in missed one or more training sessions/games. After application of the inclusion and exclusion criteria, 10 home game weeks (Table 1: game weeks 7, 10, 14, 16, and 20 using Model I; game weeks 4, 5, 12, 24, and 27 using Model II) and twenty-six players were included.

**Table 1:** Organization of the Pro D2 season and the arrangement of the two morphocycle models.

	Professional Rugby Union Season																																							
Months	July	August		September				October				November					December				January					February				March				April				May		
**Periods**	Pre-season			1^st^ block				2^nd^ block					Rest	Rest	3^rd^ block					Rest	Rest	4^th^ block				Rest	5^th^ block					Rest	6^th^ block				Rest	7^th^ block		
**Game Weeks**		FG	FG	1	2	3	4	5	6	7	8	9			10	11	12	13	14			15	16	17	18		19	20	21	22	23		24	25	26	27		28	29	30
**Training Models**				M2	M1	-	M2	M2	-	M1	M2	M1			M1	M1	M2	M2	M1			M1	M1	M2	-		-	M1	M2	-	-		M2	M2	-	M2		-	-	-
**Game Place**		A	H	H	A	A	H	H	A	H	A	A			H	A	H	A	H			A	H	A	H		A	H	A	H	A		H	A	H	H		A	H	A
**Game Results**		△	○	○	△	○	○	○	○	○	○	○			△	○	○	□	○			○	○	○	○		○	□	○	○	○		○	△	○	○		△	△	△

M1 = Model I; M2 = Model II; - = other training models; FG = friendly game; A = away game; H = home game; ○= win; □= draw; △= defeat; Different tones of gray markers shading represent the models and the competition weeks selected for data analysis.

Reprinted from [Xiaopan Hu, Rugby Club Vannes] under a CC BY license, with permission from [Rugby Club Vannes], original copyright [2020-2021].

Twenty-six adult rugby union players (15 forwards, 11 backs), all male (mean ± standard deviation [SD]; age 27 ± 3.5 years; height 185.6 ± 7.1 cm; body mass 101.7 ± 15.7 kg) and members of a professional club playing in the French second division (Pro D2), participated in this study. All participants were informed of the experimental protocols and potential risks and benefits of the study. They were subsequently required to complete and sign a consent form indicating their permission for their data to be utilized in the study. The protocol was approved by the Research Ethics Committee of the University of Rennes and the professional rugby club and conducted in accordance with the Declaration of Helsinki.

### Procedures

All participants routinely engaged in gym sessions twice per week and on-field training sessions thrice per week. Before on-field training on the StD and EnD, participants completed gym sessions (approx. 45 mins). The on-field training included a collective drill and several distinct overarching training focuses. Overall, each participant completed 50 training sessions (20 gym and 30 on-field sessions) under the observational research protocol. The monitoring period of each session included the warm-up and main training phases. No data were obtained from the rehabilitation, recuperation, or cool down phases. We did not control for variables such as weather or competitive match results.

#### Training session management

The order in which acquisition days are scheduled and their consequent efficiency are dependent on the principle of specificity and a series of rugby-specific factors [7]. They target each of the three main physical fitness (Fig 1). The StD was always scheduled four days before the match day to allow maximum recovery between the last match and the next. As the first acquisition day in both models, the StD emphasized anaerobic conditions that typically aim to overload acceleration, deceleration, and change of direction efforts through confined playing spaces with contact-focused technical or tactical activities [15, 16]. In addition to small-sided games (SSGs; e.g., 20 × 30 m) configured in small or medium number of players (e.g., 5 *vs*. 5 or 4 *vs*. 3), condition exercises such as wrestling, sled push, and sled sprints were used to create the second-half fatigue effect.

The EnD occurred on a central day, though its placement varied between the models. It was designed with a wide space (e.g., 50 × 60 m or full field), a greater number of players (e.g., 10 *vs*. 10 or 10 *vs*. 8), and more movement to encourage adaptation to a playing style based specifically on endurance [7]. The demands of the conditioning session resembled those of a real match, and the tactical contexts were based on the features of the next opposing team to stimulate collective interactions. There are two reasons for arranging the non-contact work capacity session on the EnD. First, it has been demonstrated that external and internal workload increase during contact training and result in a reduction in upper-body neuromuscular function [7]. This may suggest that planning the rest day after the StD (as in Model I) is effective, to allow adequate recovery time to avoid decreased tackling ability. Second, the running demands in rugby union are relatively low and these can be achieved and exceeded relatively easily during field-based training in the absence of contact [7]. Thus, scheduling the EnD after the StD (as in Model II) would help maintain aerobic ability while simultaneously developing skills and tactical awareness.

To substantiate our tactical periodization training model for rugby players, a comparative design was implemented, which targeted the players’ physiological characteristics and the principal of horizontal alternation in specificity [4, 7]. In this study, the main difference between the two models is the scheduling of the rest day: before the EnD in Model I and before the SpD in Model II. Thus, we named Model I “specific endurance” and Model II “specific speed” to describe their distinctive features. For effective training, specific acquisition days and the rest day must be determined by the coach. From a physiological and biological standpoint, the programming of complete rest before a specific acquisition day aims to achieve the targeted training stimulus for that acquisition day [4, 16, 17]. Therefore, we designed the morphocycle format variation for the competitive season to progressively prepare the players according to the prevailing system of conducting competitions.

In both models, Thursday was considered as the SpD, which focused on the speed of both decision-making and movement. The on-field session should stimulate an anaerobic state among players but only for a short duration. During this session, players needed to complete most of their maximum velocity running in a medium space (e.g., 40 × 30 m) with a medium number of players (e.g., 8 *vs*. 4) [7]. As the last of the three acquisition days of the morphocycle and the final day before the game, the number of repetitions of each exercise was lower on the SpD than on the StD and EnD to decrease the overall mental emotional and physical strain [13]. Since the SpD followed different structural units (after the EnD in Model I, after the rest day in Model II), it is important to clarify that the training tasks were not the same under both models.

#### External workload monitoring

The external workload was assessed using a portable 10 Hz GPS unit (Vector X7, Catapult Sports^®^, Australia). The reliability (coefficient of variation [CV] 1.9‒6.0%) and accuracy (3.1‒11.3%) of this equipment have been evaluated in many studies [18, 19]. About 20 min before the on-field training, devices were turned on and left in an open area to attain satellite connection. Players wore individual GPS units (81 mm × 43 mm × 16 mm, weighing 53 g) that were placed in a custom-designed pocket positioned between the shoulder blades on a vest supplied by the Catapult company.

The variables collected using GPS are described in Table 2. All these data were downloaded immediately after every field training session and analyzed using customized software (Open Field, version 3.3, Catapult, Melbourne, Australia).

**Table 2:** Global positioning system variables used to quantify external workload.

Variables	Units	Description
**PlayerLoad**^**™**^ **(PL**^**™**^**)**	Arbitrary units	A modified vector magnitude expressed as the square root of the sum of the squared instantaneous rates of change in acceleration in each of the three orthogonal planes and divided by 100 [20].
**PlayerLoad**_**slow**_ **(PL**_**slow**_**)**	Arbitrary units	A vector magnitude represented activity from three planes of motion, but only for movement below 2 m·s^-1^ [20].
**Total distance (TD)**	Meters	Assessed from GPS, correspond to the total distance covered by the players during the ball-in-play time of training.
**High-speed running (HSR)**	Meters	Sum of distance covered above 15 km·h^-1^.
**Very high-speed running (VHSR)**	Meters	Sum of distance covered above 21 km·h^-1^.
**Sprint running (SR)**	Meters	Sum of distance covered above 25 km·h^-1^.
**Repeated high-intensity efforts (RHIE)**	Number	Three consecutive high-intensity efforts (contact, acceleration, or sprint) occurring within 21 seconds [18].
**Low acceleration and deceleration ([A-D]2)**	Number	Number of accelerations and decelerations performed above 2 m·s^-2^ or below -2 m·s^-2^.
**Medium acceleration and deceleration ([A-D]2.5)**	Number	Number of accelerations and decelerations performed above 2.5 m·s^-2^ or below -2.5 m·s^-2^.

#### Internal workload monitoring

The internal workload was estimated using the s-RPE on a 1–10 arbitrary unit rating scale, with a rating of 0 deemed as rest and 10 as the maximal perceived effort [21]. s-RPE data were recorded in all on-field sessions for which GPS devices were used. Approximately 30 min after each on-field session, the researchers recorded the players individually and asked them how difficult they felt the session was following the subjective scale of Foster et al. [21]. The answer was subsequently multiplied by the session duration (in minutes) to obtain the s-RPE. The duration of the session was timed by the coach, from the start of the warm-up to the completion of the last training drill. Data for s-RPE are presented as arbitrary units (AU).

### Statistical analyses

All data are expressed as mean ± SD. For the analysis of the results, SPSS (IBM Inc, Chicago, IL), version 25.0 was used. The normality of the data distribution was verified using the Shapiro—Wilk test, and homoscedasticity was confirmed using Levene’s test. We conducted a three-way ANOVA analysis to test the morphocycle models, acquisition days, and playing position effects. The effects of tactical periodization training on the dependent variables were tested using repeated measures analysis of variance, with Bonferroni post hoc. Student’s *t*-test was used to determine differences between forwards and backs for all data. The alpha level for statistical significance was set at *p*<0.05. Significant differences were further analyzed using the effect size (ES), classified according to Cohen’s rule of thumb (<0.20, trivial; 0.21–0.59, small; 0.60–1.19, moderate; 1.20–2.00, large; and >2.00, very large) [22].

## Results

### External workload

#### External workload differences between models

The external workload values of all participants in the two tactical periodization models are presented in Tables 3 and 4. For both positions, on the StD, players showed significantly greater high-speed running (HSR) and very high-speed running (VHSR) in Model I than Model II, whereas they showed higher PL_slow_ under Model II. Moreover, the StD also had a considerably larger PlayerLoad^™^ (PL^™^; *p*<0.05, ES: 0.3) and number of accelerations and decelerations ([A-D]2; *p*<0.05, ES: 0.3) for forwards and a higher sprint running (SR; *p*<0.05, ES: 0.4) for backs in Model I. Concerning the EnD, five weeks of Model II training stimulated more external workload among forwards, reflected by PL^™^ (*p*<0.05, ES: -0.4), total distance (TD; *p*<0.01, ES: -0.4), and [A-D]2 (*p*<0.01, ES: -0.5). However, no significant differences were found in backs’ external workload parameters on EnD between the two models (*p* = 0.07‒0.98). Results from the SpD further revealed the morphocycles’ characteristics, namely that all external workload were higher in Model II than in Model I for both positional units, except forwards’ VHSR (*p* = 0.18) and SR (*p* = 0.72).

**Table 3:** Mean ± standard deviation of the external load variables depending on morphocycle models, acquisition days, and playing positions during 10 in-season weeks.

	Model I						Model II					
	Strength		Endurance		Speed		Strength		Endurance		Speed	
Variable	Forwards	Backs	Forwards	Backs	Forwards	Backs	Forwards	Backs	Forwards	Backs	Forwards	Backs
**PL**^**™**^ **(AU)**	381.0 ± 74.4	465.0 ± 79.2*	358.8 ± 64.5^a^	410.3 ± 72.1^a^*	183.5 ± 26.7^a^^b^	192.8 ± 36.8^a^^b^	359.7 ± 54.6^†^	462.4 ± 82.9*	382.8 ± 65.8^†^^a^	413.7 ± 73.3^a^*	240.1 ± 36.0^†^^a^^b^	274.2 ± 52.7^†^^a^^b^*
**PL**_**slow**_ **(AU)**	153.4 ± 31.9	160.1 ± 28.3	150.3 ± 26.7	131.4 ± 25.4^a^*	83.9 ± 13.8^a^^b^	76.2 ± 15.6^a^^b^	177.8 ± 28.6^†^	179.3 ± 30.0^†^	154.9 ± 28.1^a^	134.8 ± 30.6^a^*	103.4 ± 21.4^†^^a^^b^	102.7 ± 24.9^†^^a^^b^
**TD (m)**	3292.0 ± 631.4	4454.1 ± 671.3*	3203.8 ± 476.5	3978.5 ± 561.2^a^*	1730.2 ± 282.2^a^^b^	1884.2 ± 316.7^a^^b^	3187.5 ± 564.3	4394.6 ± 700.0*	3431.7 ± 599.8^†^^a^	4018.2 ± 645.2^a^*	2174.3 ± 208.4^†^^a^^b^	2541.6 ± 304.4^†^^a^^b^*
**HSR (m)**	471.0 ± 222.9	877.7 ± 241.8*	432.4 ± 168.6	848.1 ± 221.0*	248.6 ± 199.4^a^^b^	315.6 ± 136.0^a^^b^	327.7 ± 183.0^†^	747.5 ± 263.1^†^*	479.5 ± 213.6^a^	847.2 ± 251.3^a^*	315.1 ± 110.3^†^^b^	512.4 ± 174.6^†^^a^^b^*
**VHSR (m)**	70.7 ± 71.8	196.8 ± 97.4*	63.0 ± 82.0	228.0 ± 122.9^a^*	21.9 ± 28.5^a^^b^	76.1 ± 63.6^a^^b^*	42.1 ± 56.6^†^	160.3 ± 98.1^†^*	70.3 ± 78.6^a^	215.9 ± 111.2^a^*	40.3 ± 40.6^b^	138.8 ± 122.4^†^^b^*
**SR (m)**	7.6 ± 14.6	32.7 ± 27.5*	7.8 ± 15.6	52.1 ± 50.8^a^*	2.0 ± 6.8	13.3 ± 25.9^a^^b^*	4.7 ± 9.8	23.0 ± 22.9^†^*	11.5 ± 21.2	43.7 ± 36.5^a^*	3.4 ± 8.6^b^	26.8 ± 27.6^†^^b^*
**RHIE (n)**	6.1 ± 4.6	15.5 ± 6.2*	5.2 ± 3.2	14.3 ± 6.4*	2.2 ± 2.1^a^^b^	3.6 ± 2.9^a^^b^	5.6 ± 4.2	14.4 ± 6.6*	6.3 ± 3.8	14.0 ± 5.9*	4.5 ± 2.7^†^^b^	7.9 ± 4.3^†^^a^^b^*
**[A-D]2 (n)**	89.8 ± 30.8	144.5 ± 30.2*	75.9 ± 18.3^a^	122.4 ± 25.8^a^*	38.4 ± 11.8^a^^b^	44.2 ± 11.3^a^^b^	81.5 ± 24.5^†^	145.7 ± 36.3*	86.0 ± 24.0^†^	129.1 ± 29.5^a^*	58.8 ± 11.7^†^^a^^b^	75.7 ± 20.4^†^^a^^b^*
**[A-D]2.5 (n)**	43.9 ± 19.9	81.6 ± 23.6*	36.3 ± 12.3^a^	70.5 ± 20.9^a^*	20.2 ± 8.1^a^^b^	23.8 ± 8.4^a^^b^	42.4 ± 16.5	86.0 ± 26.1*	41.2 ± 14.0	72.4 ± 22.5^a^*	31.8 ± 8.2^†^^a^^b^	43.3 ± 14.2^†^^a^^b^*

PL^™^ = PlayerLoad^™^; PL_slow_ = PlayerLoad_slow_; TD = total distance; HSR = high-speed running; VHSR = very high-speed running; SR = sprint running; RHIE = repeated high-intensity efforts; [A-D] = number of accelerations and decelerations.

^†^significant differences between Model I and Model II, *p*<0.05;

^a^ significant difference with the values of the strength day, *p*<0.05;

^b^ significant difference with the values of the endurance day, *p*<0.05;

*significant differences between forwards and backs, *p*<0.05.

**Table 4:** *P* values and effect sizes of the workload variables depending on morphocycle models, acquisition days, and playing positions during 10 in-season weeks.

Comparison		F-StD-M1 *vs*. M2	F-EnD-M1 *vs*. M2	F-SpD-M1 *vs*. M2	B-StD-M1 *vs*. M2	B-EnD-M1 *vs*. M2	B-SpD-M1 *vs*. M2	M1-F-StD *vs*. EnD	M1-F-StD *vs*. SpD	M1-F- EnD *vs*. SpD	M2-F-StD *vs*. EnD	M2-F-StD *vs*. SpD	M2-F- EnD *vs*. SpD	M1-B-StD *vs*. EnD	M1-B-StD *vs*. SpD	M1-B- EnD *vs*. SpD	M2-B-StD *vs*. EnD	M2-B-StD *vs*. SpD	M2-B- EnD *vs*. SpD	M1-StD-F *vs*. B	M1-EnD-F *vs*. B	M1-SpD-F *vs*. B	M2-StD-F *vs*. B	M2-EnD-F *vs*. B	M2-SpD-F *vs*. B
Variables																									
**PL**^**™**^ **(AU)**	***p***	0.03	0.17	0.00	0.83	0.77	0.00	0.03	0.00	0.00	0.02	0.00	0.00	0.00	0.00	0.00	0.00	0.00	0.00	0.00	0.00	0.40	0.00	0.01	0.00
	**ES**	0.3	-0.4	-1.8	NS	NS	1.8	0.3	3.5	3.6	-0.4	2.6	2.7	0.7	4.4	3.8	0.6	2.7	2.2	-1.1	-0.8	NS	-1.5	-0.4	-0.8
**PL**_**slow**_ **(AU)**	***p***	0.00	0.27	0.00	0.00	0.50	0.00	0.46	0.00	0.00	0.00	0.00	0.00	0.00	0.00	0.00	0.00	0.00	0.00	0.15	0.00	0.92	0.74	0.00	0.87
	**ES**	-0.8	NS	-1.1	0.7	NS	-1.3	NS	2.8	3.1	0.8	3.0	2.1	1.1	3.7	2.6	1.5	2.8	1.2	NS	0.7	NS	NS	0.7	NS
**TD (m)**	***p***	0.22	0.01	0.00	0.55	0.69	0.00	0.30	0.00	0.00	0.00	0.00	0.00	0.00	0.00	0.00	0.00	0.00	0.00	0.00	0.00	0.10	0.00	0.00	0.00
	**ES**	NS	-0.4	-1.8	NS	NS	-2.1	NS	3.2	3.8	0.4	2.4	2.8	0.8	4.9	4.6	0.6	3.4	2.9	-1.8	-1.5	NS	-1.9	-0.9	-1.4
**HSR (m)**	***p***	0.00	0.15	0.04	0.00	0.98	0.00	0.24	0.00	0.00	0.00	0.70	0.00	0.44	0.00	0.00	0.01	0.00	0.00	0.00	0.00	0.06	0.00	0.00	0.00
	**ES**	0.7	NS	-0.4	0.5	NS	-1.3	NS	1.1	1.0	-0.8	NS	1.0	NS	2.9	2.9	-0.4	1.1	1.6	-1.8	-2.1	NS	-1.9	-1.6	-1.4
**VHSR (m)**	***p***	0.04	0.59	0.18	0.02	0.45	0.00	0.57	0.00	0.00	0.04	0.89	0.03	0.05	0.00	0.00	0.00	0.18	0.00	0.00	0.00	0.00	0.00	0.00	0.00
	**ES**	0.4	NS	NS	0.4	NS	-0.6	NS	0.9	0.7	-0.4	NS	0.5	0.3	1.5	1.6	-0.5	NS	0.7	-1.5	-1.6	-1.1	-1.5	-1.5	-1.1
**SR (m)**	***p***	0.46	0.35	0.72	0.03	0.07	0.00	0.95	0.16	0.14	0.08	0.74	0.04	0.00	0.00	0.00	0.00	0.40	0.00	0.00	0.00	0.01	0.00	0.00	0.00
	**ES**	NS	NS	NS	0.4	NS	-0.5	NS	NS	NS	NS	NS	0.5	0.5	0.7	1.0	0.7	NS	0.5	-1.1	-1.2	-0.6	-1.0	-1.1	-1.2
**RHIE (n)**	***p***	0.52	0.12	0.00	0.22	0.80	0.00	0.22	0.00	0.00	0.34	0.15	0.02	0.15	0.00	0.00	0.64	0.00	0.00	0.00	0.00	0.09	0.00	0.00	0.00
	**ES**	NS	NS	-1.0	NS	NS	-1.2	NS	1.1	1.1	NS	NS	0.6	NS	2.5	2.2	NS	1.2	1.2	-1.7	-1.8	NS	-1.6	-1.6	-0.9
**[A-D]2 (n)**	***p***	0.03	0.01	0.00	0.80	0.14	0.00	0.00	0.00	0.00	0.25	0.00	0.00	0.00	0.00	0.00	0.00	0.00	0.00	0.00	0.00	0.17	0.00	0.00	0.00
	**ES**	0.3	-0.5	-1.7	NS	NS	-1.9	0.6	2.2	2.4	NS	1.2	1.4	0.8	4.4	3.9	0.5	2.4	2.1	-1.8	-2.1	NS	-2.1	-1.6	-1.0
**[A-D]2.5 (n)**	***p***	0.58	0.07	0.00	0.17	0.55	0.00	0.01	0.00	0.00	0.68	0.00	0.00	0.00	0.00	0.00	0.00	0.00	0.00	0.00	0.00	0.22	0.00	0.00	0.00
	**ES**	NS	NS	-1.4	NS	NS	-1.7	0.5	1.6	1.6	NS	0.8	0.8	0.5	3.3	2.9	0.6	2.0	1.6	-1.7	-2.0	NS	-2.0	-1.7	-1.0

F = forwards; B = backs; StD = strength day; EnD = endurance day; SpD = speed day; M1 = Model I; M2 = Model II; *p* = *p* value; ES = effect size; PL^™^ = PlayerLoad^™^; PL_slow_ = PlayerLoad_slow_; TD = total distance; HSR = high-speed running; VHSR = very high-speed running; SR = sprint running; RHIE = repeated high-intensity efforts; [A-D] = number of accelerations and decelerations.

#### External workload differences among acquisition days

Pairwise comparisons between acquisition days under the same model and position are reported in Tables 3 and 4. In Model I, the StD was characterized by more PL^™^, [A-D]2, and [A-D]2.5 than the EnD and SpD for both positions. In Model II, the StD had a peak PL_slow_ value and the highest HSR and VHSR occurred on the EnD for both forwards and backs. Moreover, both morphocycle models presented similar distributions in that external workload decreased most significantly on the SpD. In addition, in each position, there were significant differences among morphocycle acquisition days for some variables. For example, backs had a significantly higher PL_slow_ and TD on the StD and a significantly higher VHSR and SR on the EnD in Model I. In Model II, backs had a higher PL^™^, TD, [A-D]2, and [A-D]2.5 on the StD, as well as a greater SR on the EnD. There was also a significantly higher PL^™^ and TD for forwards on the EnD in Model II.

#### External workload differences between positions

Tables 3 and 4 highlight that on the StD, backs in both models showed greater external workload values than forwards (*p*<0.001; ES: 1.0‒2.8), except for PL_slow_ (*p*>0.05). This trend was also evident across all external workload variables (*p*<0.001; ES: 0.4‒2.1) on the EnD except PL_slow_ for which forwards scored higher (*p*<0.001; ES: 0.7) than backs. However, it was observed that, in the case of the SpD in Model I, backs only covered a greater VHSR and SR (*p*<0.001; ES: 0.6‒1.1) than forwards. Finally, backs’ external workload parameters were significantly greater (*p*<0.001; ES: 0.8‒1.4) than those of in forwards on the SpD in Model II, except for PL_slow_ (*p*>0.05).

### Internal workload

#### Internal workload differences between models

**Fig 2:**
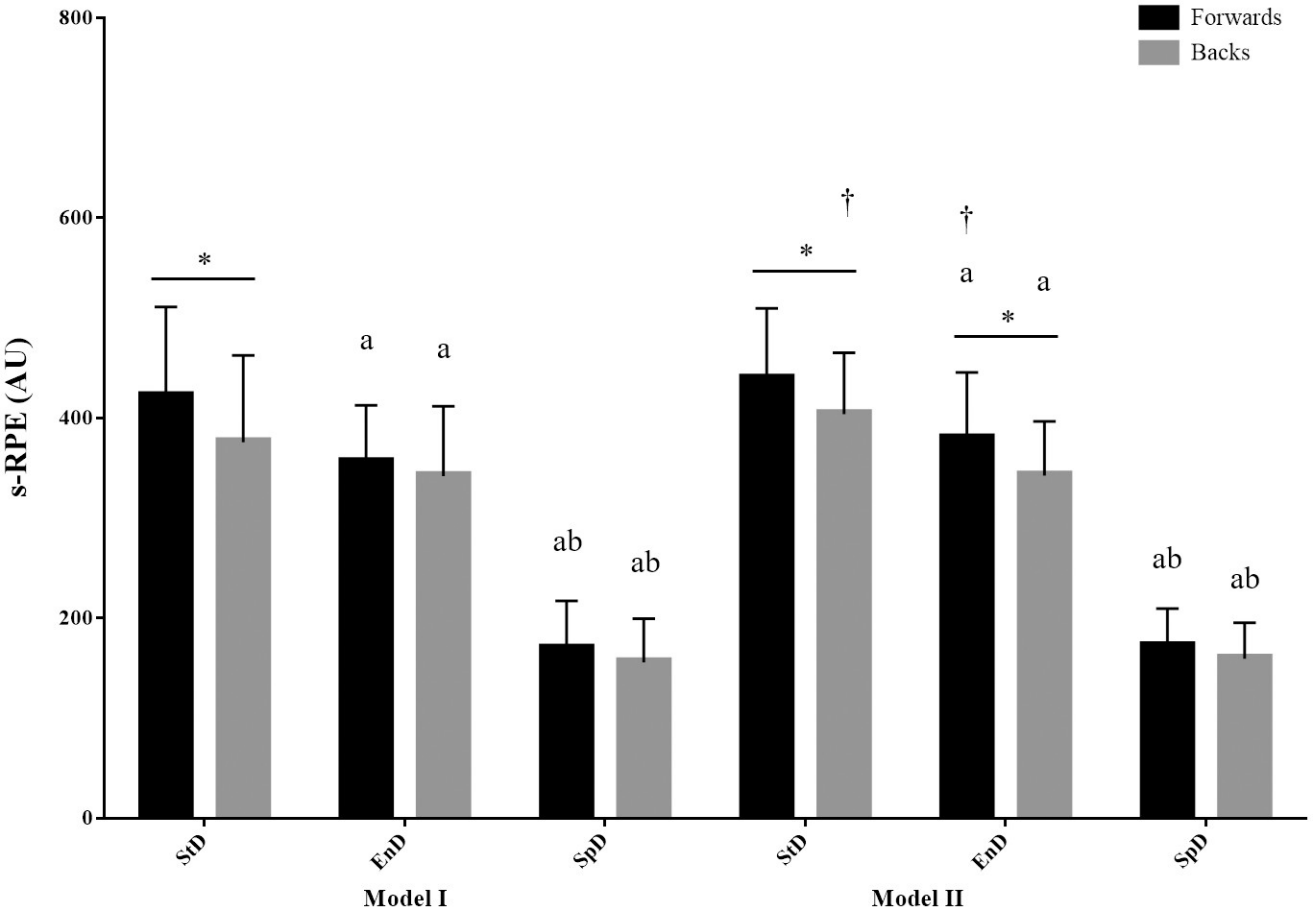
Mean and standard deviation of session rating of perceived exertion between positions across the three acquisition days in the two tactical periodization models. s-RPE = session rating of perceived exertion; AU = arbitrary units; StD = strength day; EnD = endurance day; SpD = speed day. ^†^significant differences between Model I and Model II, *p*<0.05; ^a^ significant difference with the values of the strength day, *p*<0.05; ^b^ significant difference with the values of the endurance day, *p*<0.05; *significant differences between forwards and backs, *p*<0.05.

Cumulative internal workload values are displayed in Fig 2, showing inclusive comparisons of the models, acquisitions days, and positions. When comparing the models, significant differences were identified for backs on the StD (Model I *vs*. Model II, *p*<0.05, ES: -0.4) and for forwards on the EnD (Model I *vs*. Model II, *p*<0.05, ES: -0.4); overall, Model II had a higher internal workload response than Model I.

#### Internal workload differences among acquisition days

When comparing acquisition days (Fig 2), the mean s-RPE was significantly altered during three acquisitions days in two models with the highest values on the StD (Model I: forwards: 422.0 ± 89.1 AU, backs: 375.9 ± 86.8 AU; Model II: forwards: 439.4 ± 70.3 AU, backs: 404.1 ± 61.3 AU) and the lowest values on the SpD (Model I: forwards: 169.4 ± 47.8 AU, backs: 155.8 ± 43.6 AU; Model II: forwards: 171.9 ± 37.7 AU, backs: 159.4 ± 36.1 AU) for both positions (Model I: forwards: [StD *vs*. EnD, *p*<0.001, ES: 0.9], [StD *vs*. SpD, *p*<0.001, ES: 3.5], [EnD *vs*. SpD, *p*<0.001, ES: 3.6]; backs: [StD *vs*. EnD, *p*<0.01, ES: 0.4], [StD *vs*. SpD, *p*<0.001, ES: 3.2], [EnD *vs*. SpD, *p*<0.001, ES: 3.2]; Model II: forwards: [StD *vs*. EnD, *p*<0.001, ES: 0.9], [StD *vs*. SpD, *p*<0.001, ES:4.7], [EnD *vs*. SpD, *p*<0.001, ES: 3.9]; backs: [StD *vs*. EnD, *p*<0.001, ES:1.1], [StD *vs*. SpD, *p*<0.001, ES: 4.9], [EnD *vs*. SpD, *p*<0.001, ES: 4.0]).

#### Internal workload differences between positions

Analysis of the difference between positions showed that forwards’ s-RPE on the StD (Model I: *p*<0.001, ES: 0.5; Model II: *p*<0.01, ES: 0.5) in both the models and on the EnD in Model II was higher (*p*<0.01, ES: 0.6) than that of backs (Fig 2).

## Discussion

Tactical periodization is becoming more popular worldwide, notably in rugby union [7]. However, scientific research has yet to catch up with practice and provide empirical support for its expansion. Furthermore, the workload on typical acquisition days completed by different position units in rugby union has not been thoroughly researched [2]; thus the optimal application of tactical periodization models to fulfil the demands of in-season rugby union training is poorly understood. Therefore, we present the first study to describe external and internal workload profiles and compare position differences among professional rugby union players across three acquisition days under two types of tactical periodization models. As hypothesized, we observed substantial differences between forwards and backs during the tactical periodization training period. Furthermore, the results suggest that Model I, named “specific endurance”, induces more aerobic running in parameters relevant to endurance performance, whereas Model II, named “specific speed”, requires maintaining the necessary intensity on the SpD to improve speed performance. Additionally, we observed a tapering strategy from the first two acquisition days (StD and EnD) to the final acquisition day (SpD) in both models. Finally, the findings highlight the importance of considering positional differences when developing tactical periodization-based training programs. In summary, these data provide new information for applied tactical periodization in rugby union and can be used to design specific morphocycle models.

### Effect of the morphocycle models on external and internal workload

#### External workload

To the best of our knowledge, this is the first study to examine the application of two tactical periodization morphocycle models for the in-season training of a professional rugby union team, utilizing a workload monitoring protocol. For all external workload variables, trivial to very large ES between Model I and Model II were identified. These differences were evident even though the study protocol analyzed the effect of only five weeks of each morphocycle model on already well-trained rugby union players.

According to the rugby union physical development framework, the ideal training plan should integrate various training program elements, such as strength, endurance, speed, and recovery [23]. Simply adopting the same weekly training pattern to prepare for the entire season is a limiting strategy. However, much of the current strength and conditioning literature fails to address the issue of how different tactical periodization training structures may influence workload response, owing to the use of a single-model [5, 6]. Thus, in this study, team-certified and highly experienced coaches designed two morphocycle models based on the tactical periodization concept, containing three acquisition days targeting three main physical capacities. As noted in the procedures, Model I emphasized specific endurance development, whereas Model II emphasized specific speed activities. Their objectives are accordingly reflected by the GPS results (Tables 3 and 4). First, Model I achieved the greatest HSR and VHSR for both positions on the StD. In line with the findings on the association between aerobic fitness and key running attributes [24, 25], these results confirm that Model I was indeed an endurance-oriented morphocycle. Therefore, sufficient stimulus for aerobic adaptation was achieved, as demonstrated by absolute HSR and VHSR performance, meeting the requirements of higher competition levels that apply to all players. Second, the EnD in Model I was followed by the SpD and the match day, whereas the EnD in Model II was scheduled between the StD and the rest day. Both the models revealed a similar external workload, except for PL^™^, TD, and [A-D]2 for forwards (Tables 3 and 4). Based on these results, it can be assumed that players on the EnD in both models were exposed to a similar overload volume, which induced maximal adaptation without increasing the risk of accumulated fatigue [23]. Furthermore, PL^™^, TD, and [A-D]2 may be effective external workload variables for comparing elite players’ responses to training between positions [26]. Finally, the external workload parameters were higher in Model II. As a speed-oriented morphocycle, in Model II, players (especially backs) accomplished more accelerations, distances and relatively high-intensity running distances. These attributes are considered important in rugby, which consists of multiple intermittent high-intensity activities [14, 24]. It should be noted that the SpD allocates one-quarter of the session for speed of execution and three-quarters for recovery in Model I. This acquisition day aims to achieve the desired external workload that stimulates players to remain at an optimal level of physical fitness [13, 27]. By contrast, the SpD in Model II was scheduled after the rest day; the recovery day prior to the SpD aimed maximize the effect of speed in terms of muscle fiber contractions [13]. Differences in the rest day arrangement and acquisition day objectives likely influenced the morphocycle and positional external workload variation.

#### Internal workload

The findings revealed a significant difference in internal workload response between Model I and Model II on the StD for backs and the EnD for forwards (Fig 2). In recent years, researchers have become increasingly interested in the relationship between internal and external workload measures in team sports. Studies have demonstrated that HSR, accelerations and decelerations are the best predictors of s-RPE during team sports [15, 28]. Research has also shown a clear association between s-RPE with PL^™^ [29] and TD [28]. Therefore, the possible explanations of the s-RPE observed in the present study could be the accumulated high-intensity running for backs on the StD in Model I and the higher PL^™^, TD, and [A-D]2 for forwards on the EnD in Model II. According to the characteristics of the next opponent, the morphocycle alternation respected the methodological principle of stabilization to achieve optimal performance over a long competitive period. Logically, the internal workload would be influenced by the training philosophy. Understanding the variability of internal workload data is crucial in practice as it helps practitioners evaluate whether a change in workload between morphocycle is meaningful. Moreover, understanding the effect of two tactical periodization models on internal workload in rugby union can assist all staff in efficiently transferring theory to practice. This study further supports the concept that s-RPE can be a useful tool for monitoring internal workload in rugby union training.

### Effect of acquisitions days on external and internal workload

#### External workload

In both the morphocycle models, we observed clear evidence of external workload periodization variation in the acquisition days leading up to game day. Mujika and Padilla [30] defined this period as the taper, that is, “a systematic, nonlinear reduction of workload during a variable period to reduce the physiological and psychological stress from daily training and optimize sports performance”. Among the three acquisition days in the two tactical periodization models (Tables 3 and 4), the similar external workload pattern from the StD to SpD in both models indicates the management of the contents during the week based on classic biological laws [13]. Generally, rugby union players have two rest days during the six-day morphocycle, the first day being scheduled the day after the match [31]. Therefore, we proposed that the second rest day be scheduled between the StD and EnD (Model I) or the EnD and SpD (Model II) to provide a minimum of s 24-hour recovery period from high-intensity or high-volume training. In the present study, all external workload variables significantly lower on the SpD than the StD and EnD, except for some specific position-related parameters (e.g., forwards’ SR on the SpD in Model I). Similar results were reported for elite soccer [32], ice hockey [33], and among six professional rugby union teams [34]. Our findings confirmed that there is an obvious external workload pattern across acquisition days in the lead-up to a game day. In this regard, the SpD incorporates tapering to improve players’ match readiness.

#### Internal workload

The internal workload reported in the current study also indicates a progressive reduction of workload during the week and before the competition (Fig 2). This unloading process is comparable to that found in previous research. Malone et al. [32] reported that workload was lower on the day before the match (match day-1, MD-1) than MD-2 and MD-5. Similar unloading processes were found in elite soccer players, with MD-3 having the highest s-RPE and MD-1 containing the lowest [32]. Foster et al. [35] emphasized the importance of workload variation, stating that a lack of workload variety might lead to training monotony and strain and fail to elicit the desired adaptions. In addition to workload unloading, there is consensus that the composition of morphocycle training typology should different between collision and non-collision sports [36]. Regardless of the sport, tapering strategy should be used by coaches to limit fatigue leading up to a game. However, there is a clear logic that when considering workload arrangement, sport and position-specific roles should be considered. Overall, our internal workload results with respect to acquisition days are consistent with previous research showing that a tapering strategy was implemented before the competition to ensure optimal player readiness for match day [36].

### Effect of player positions on external and internal workload

#### External workload

Regarding position, there were significant differences in external workload on each acquisition day between forwards and backs (Tables 3 and 4). More precisely, backs’ training session contained more PL^™^, TD, distances in a different speed zone, repeated thigh-intensity efforts (RHIE), and acceleration and deceleration. By contrast, PL_slow_ presented a converse trend on the EnD, with no differences between forwards and backs on the StD and SpD. Our external workload results tend to agree with those of previous studies [20, 37, 38]. In Super 14 rugby union, Austin et al. [37] reported that inside backs spent more time (426 ± 17 *vs*. 285 ± 72 s) and covered more high-intensity running distances (1399 ± 91 *vs*. 940 ± 257 m) than front row forwards. Similarly, Cahill et al. [38] reported that backs travelled greater absolute and relative distances than forwards in English Premiership rugby union matches. Roe et al. [20], who investigated the relationship between PlayerLoad variables (PL^™^ and PL_slow_) and both collisions and running demands among elite under-18 rugby union players, reported that there are very large (nearly perfect) relationships between PL^™^ and TD for both positions. According to these authors, PL_slow_ is the most effective indicator of collision activity for forwards and backs. In our study, the higher PL_slow_ for forwards on the EnD may reflect the training specificity between positions. Furthermore, on the StD and SpD, there were no significant differences in PL_slow_ between forwards and backs, which could be due to backs covering greater distance at low velocity and thus reducing the gap with forwards [20]. However, the TD, PL^™^, and PL_slow_ observed in the current professional team were lower than those reported in Roe et al.’s [20] study. This is likely the result of our alternative method for quantifying external workload, which analyzed data from ball-in-play time (i.e., without stoppages in play [ball out of play time]) [10].

The external workload between forwards and backs on the StD and EnD did seem to have the same pattern in the two models, in which backs regularly performed higher external workload than forwards, except for the PL_slow_ metric. These findings support previous studies that have found differences between forwards and backs in training activities and match play [37–39], with forwards spending more time in the lower speed zones and engaging in a larger number of tackling, rucking, mauling and other static high-intensity actions. Backs perform multiple accelerations, decelerations, and changes of direction and reach individual maximal speed to gain territory or score points [10]. Under this condition, the absolute measure of PL_slow_ was higher for forwards than for backs on the EnD, which most likely reflects a relatively greater proportion of training drills performed at low velocities by forwards, such as contact and wrestling [40, 41]. Furthermore, Hulin et al. [41] found that PL_slow_ demonstrated the greatest sensitivity to change of direction workloads when movement velocities were below 2 m·s^-1^. Previous research has also suggested that vertical accelerometer measurements make a greater contribution to PL^™^ for backs [40]. These findings provide empirical evidence explaining the lack of difference in PL_slow_ between forwards and backs on the StD and SpD in both models.

Additionally, also of interest were the external workload differences between forwards and backs when comparing the SpD between the two models. On the SpD in Model I, positional differences only occurred in VHSR, SR, and RHIE, whereas all external workload metrics (except for PL_slow_) presented positional differences on the SpD in Model II. These results revealed that the tactical periodization design satisfied the need to accommodate greater high-intensity running by backs on the SpD. Based on previous results relating to five different field sports, the distance covered in differentiated speed zones relates to the typical locomotive activity profiles of intermittent team sports [42]. Our data demonstrate the importance of using absolute speed thresholds and RHIE bouts to distinguish the playing demands of different player positions in rugby union. In addition, tactical periodization methods utilized in rugby union will influence positional external workload outputs. Future research could consider using individual thresholds to describe players’ performance and workload. Finally, the positional differences of external workload metrics on the SpD between two models can be explained by the different rest day arrangement.

#### Internal workload

Considering the positional internal workload differences in the current study, forwards had greater internal workload compared to backs on the StD in Model I (422.0 ± 89.1 *vs*. 375.9 ± 86.8 AU) and Model II (439.4 ± 70.3 *vs*. 404.1 ± 61.3 AU; Fig 2). A similar trend was observed in the analysis of the internal workload of the EnD in Model II, where forwards (379.8 ± 65.9 AU) exhibited higher s-RPE than backs (342.5 ± 54.2 AU). The internal workload characteristics of forwards and backs shown in Fig 2 agree with those in previous studies [18, 27]. For instance, Bradley et al. [18]. investigated 45 elite European rugby union players and found a significantly greater s-RPE for forwards (3398 ± 335 AU) compared with backs (2944 ± 410 AU) despite lower external workload scores among forwards (e.g., TD: 9974 ± 1404 m) than backs (11585 ± 1810 m). Additionally, Barnard et al. [43]. reported that forwards (3311 ± 939 AU) had higher internal workload than backs (2851 ± 1080 AU) over an 11-week in-season period. These data suggest that the relatively static isometric but high-intensity activities performed during forward-specific training can be reflected by s-RPE. The current study has the potential to significantly add to practitioners’ understanding of the positional internal workload responses to different acquisition days. Understanding specific positional internal workload responses allows coaches, trainers, and strength and conditioning professionals to construct tactical periodization training regimens based on position-specific demands, creating consistent peak performance and decreasing injury risk. Questions remain about whether sessions consisting of various drills assessed by players as having similar intensity are equivalent in terms of workload. For example, forwards and backs may assign similar RPE scores to warm-up, SSGs, and contact training drills, but the energy expenditure and recovery time may have very different acute physiological effects and result in different training adaptations among players [44, 45].

### Limitations

While our data provide a starting point for the comprehension of tactical periodization application in rugby union, there are some general limitations that need to be acknowledged and may guide future research. First, we encourage future research to re-examine of our method using a larger sample size as this study was conducted with a single rugby union club. Second, the current investigation only used data from 10 weeks of home games, which may have potentially affected the statistical significance of the results. Future research should aim to consider home and away games together over more seasons. While acknowledging these limitations, we believe that our novel findings support the tactical periodization approach and provide original data that are especially relevant with regard to the position-specific workload characteristics observed.

### Practical applications

Tactical periodization is a training strategy that emphasizes the tactical aspects of competitive matches. It originated in soccer and is quickly gaining traction in other team sports, particularly rugby union. Rugby union players differ in external and internal workload response during training depending on their position. Such data can be used by strength and conditioning staff to understand the effect of tactical periodization on the external and internal workload; and they can facilitate the optimal application of theoretical knowledge to the training environment.

## Conclusions

The present study is the first to attempt to quantify the external and internal workload characteristics of professional rugby union forwards and backs under tactical periodization. The primary finding of this study was that Model II, which emphasized specific speed, presented higher external and internal workload than Model I, which focused on specific endurance. Our study also revealed that both the models benefitted from a tapering strategy from the first two acquisition days (StD and EnD) to the last acquisition day (SpD). Moreover, the external and internal workload of forwards and backs differ on acquisition days in the different morphocycle models, especially on the StD and EnD. We conclude that this tactical periodization approach is beneficial in rugby union training. These findings have implications for practitioners in terms of understanding and applying tactical periodization and to develop the physical attributes of rugby union players based on position-specific characteristics.

## Supporting information

S1 File GPS raw data. (CSV)

S2 File RPE raw data. (CSV)

S1 Data (PDF)

S2 Data (PDF)

S1 Text (DOC)

## References

[1] NK M Junior. Periodization models used in the current sport. MOJSM. 2020 Apr 22; 4(2):27–34. doi: 10.15406/mojsm.2020.04.00090

[2] Afonso J, Bessa C, Nikolaidis PT, Teoldo I, Clemente FM. A systematic review of research on Tactical Periodization: absence of empirical data, burden of proof, and benefit of doubt. Hum Mov. 2020 May 12; 21(4):37–43. doi: 10.5114/hm.2020.95329

[3] Mangan S, Collins K, Burns C, O’Neill C. A tactical periodisation model for Gaelic football. Int J Sports Sci Coach. 2021 17(1):208–19. doi: 10.1177/17479541211016269

[4] Mendez-Villanueva A. Tactical Periodization: Mourinho’s Best-kept secret? Soccer NSCAA J. 2012 May; 5(7):28–34.

[5] Buchheit M, Lacome M, Cholley Y, Simpson BM. Neuromuscular responses to conditioned soccer sessions assessed via GPS-embedded accelerometers: Insights into tactical periodization. Int J Sports Physiol Perform. 2018 May 1; 13(5):577–83. Epub 2017/09/06. doi: 10.1123/ijspp.2017-0045 . 28872370

[6] Lopategui IG, Paulis JC, Escudero IE. Physical Demands and Internal Response in Football Sessions According to Tactical Periodization. Int J Sports Physiol Perform. 2021 Jun 1; 16(6):858–64. Epub 2021/02/25. doi: 10.1123/ijspp.2019-0829 . 33626511

[7] Tee JC, Ashford M, Piggott D. A Tactical Periodization Approach for Rugby Union. Strength Cond J. 2018 40(5):1–13. doi: 10.1519/SSC.0000000000000390

[8] Dubois R, Lyons M, Paillard T, Maurelli O, Prioux J. Influence of weekly workload on physical, biochemical and psychological characteristics in professional rugby union players over a competitive season. J Strength Cond Res. 2018 Aug; 34(2):527–46. doi: 10.1519/JSC.0000000000002741 30074967

[9] Cunniffe B, Proctor Wayne, Baker Julien S, Davies Bruce. An Evaluation of the Physiological Demands of Elite Rugby Union Using Global Positioning System Tracking Software. J Strength Cond Res. 2009 Jul; 23(4):1195–203. doi: 10.1519/JSC.0b013e3181a3928b 19528840

[10] Pollard BT, Turner AN, Eager R, Cunningham DJ, Cook CJ, Hogben P, et al. The ball in play demands of international rugby union. J Sci Med Sport. 2018 Mar 3; 21(10):1090–4. doi: 10.1016/j.jsams.2018.02.015 . 29559318

[11] Sheehan A, Malone S, Walters A, Gabbett T, Collins K. Match-play profile of elite rugby union, with special reference to repeated high-intensity effort activity (RHIE). Sport Sci Health. 2022 Nov 15; 1(1):1–10. doi: 10.1007/s11332-021-00879-9

[12] Roberts SP, Trewartha G, Higgitt RJ, El-Abd J, Stokes KA. The physical demands of elite English rugby union. J Sports Sci. 2008 Jul; 26(8):825–33. Epub 2008/06/24. doi: 10.1080/02640410801942122 . 18569548

[13] Mallo J. Complex Football: From Seirulo´s Structured Training to Frade´s Tactical Periodization (Second Edition: Revised & Updated). Sainz JM, editor. Spain: Javier Mallo Sainz; 2020 Feb. 211 p.

[14] Duthie G, Pyne D, Hooper S. Applied physiology and game analysis of rugby union. Sports Med. 2003 Sep 4; 33(13):973–91. doi: 10.2165/00007256-200333130-00003 14606925

[15] Karlsson UB. Training/match load and physical performance in an elite female football team utilizing a tactical periodization model [Master’s thesis]. Oslo, Norges: Norwegian School of Sport Sciences; 2021.

[16] Minutillo D, Rafloski R. A practical guide to tactical periodization. Kansas City: WORLD CLASS COACHING; 2015.

[17] Martín-García A, Gómez Díaz A, Bradley PS, Morera F, Casamichana D. Quantification of a professional football team’s external load using a microcycle structure. J Strength Cond Res. 2018 Dec; 32(12):3511–8. doi: 10.1519/JSC.0000000000002816 30199452

[18] Bradley WJ, Cavanagh BP, Douglas W, Donovan TF, Morton JP, Close GL. Quantification of training load, energy intake, and physiological adaptations during a rugby preseason: a case study from an elite European rugby union squad. J Strength Cond Res. 2015 Jul; 29(9):534–44. doi: 10.1519/JSC.0000000000000631 25029003

[19] Varley MC, Fairweather IH, Aughey RJ. Validity and reliability of GPS for measuring instantaneous velocity during acceleration, deceleration, and constant motion. J Sports Sci. 2012 Jan 6; 30(2):121–7. Epub 2011/11/30. doi: 10.1080/02640414.2011.627941 . 22122431

[20] Roe G, Halkier M, Beggs C, Tilla K, Jones B. The Use of Accelerometers to Quantify Collisions and Running Demands of Rugby Union Match-Play. 2016 May; 16(2):590–601. doi: 10.1080/24748668.2016.11868911

[21] Foster C, Florhaug JA, Franklin J, Gottschall L, Hrovatin LA, Parker S, et al. A new approach to monitoring exercise training. J Strength Cond Res. 2001 Mar; 15(1):109–15. doi: 10.1519/00124278-200102000-00019 11708692

[22] Cohen J. Statistical power analysis for the behavioural sciences 2nd Edition ed. New York1988 1 July 1988.

[23] Duthie GM. A Framework for the Physical Development of Elite Rugby Union Players. Int J Sports Physiol Perform. 2006 Apr; 1:2–13. doi: 10.1123/ijspp.1.1.2 19114733

[24] Reardon C, Tobin DP, Delahunt E. Application of Individualized Speed Thresholds to Interpret Position Specific Running Demands in Elite Professional Rugby Union: A GPS Study. PLOS ONE. 2015 Jul 24; 10(7):1–12. Epub 2015/07/25. doi: 10.1371/journal.pone.0133410 26208315 PMC4514747

[25] Swaby R, Jones PA, Comfort P. Relationship between maximum aerobic speed performance and distance covered in rugby union games. J Strength Cond Res. 2016 30(10):2788–93. doi: 10.1519/JSC.0000000000001375 26890968

[26] Tierney P, Blake C, Delahunt E. Physical characteristics of different professional rugby union competition levels. J Sci Med Sport. 2021 May 23. Epub 2021/06/20. doi: 10.1016/j.jsams.2021.05.009 . 34144858

[27] Parmley J, Jones B, Sawczuk T, Weaving D. A four-season study quantifying the weekly external training loads during different between match microcycle lengths in professional rugby league. PLOS ONE. 2022 Jan 31; 17(1):e0263093. Epub 2022/02/01. doi: 10.1371/journal.pone.0263093 . 35100267 PMC8803197

[28] Gaudino P, Iaia FM, Strudwick AJ, Hawkins RD, Alberti G, Atkinson G, et al. Factors influencing perception of effort (session rating of perceived exertion) during elite soccer training. Int J Sports Physiol Perform. 2015 Oct; 10(7):860–4. Epub 2015/02/12. doi: 10.1123/ijspp.2014-0518 . 25671338

[29] Vanrenterghem J, Nedergaard NJ, Robinson MA, Drust B. Training load monitoring in team sports a novel framework separating physiological and biomechanical load-adaptation pathways. Sports Med. 2017 Nov; 47:2135–42. doi: 10.1007/s40279-017-0714-2 28283992

[30] Mujika I, Padilla S. Scientific bases for precompetition tapering strategies. Med Sci Sports Exerc. 2003 Aug; 35(7):1182–7. Epub 2003/07/04. doi: 10.1249/01.MSS.0000074448.73931.11 . 12840640

[31] Hartwig TB, Naughton G, Searl J. Load, stress, and recovery in adolescent rugby union players during a competitive season. J Sports Sci. 2009 Aug; 27(10):1087–94. Epub 2009/10/23. doi: 10.1080/02640410903096611 . 19847692

[32] Malone JJ, Michele RD, Morgans R, Burgess D, Morton JP, Drust B. Seasonal training-load quantification in elite English premier league soccer players. Int J Sports Physiol Perform. 2015 Nov 14; 10(4):489–97. Epub 2014/11/14. doi: 10.1123/ijspp.2014-0352 . 25393111

[33] Allard P, Martinez R, Deguire S, Tremblay J. In-season session training load relative to match load in professional ice hockey. J Strength Cond Res. 2022 Feb 12; 36(2):486–92. doi: 10.1519/JSC.0000000000003490 31996615

[34] West SW, Williams S, Tierney P, Batchelor T, Cross MJ, Kemp SPT, et al. Training and match load in professional rugby union: Do contextual factors influence the training week? SA J Sports Med. 2021 May; 33(1):1–6. doi: 10.17159/2078-516X/2021/v33i1a9509 36816908 PMC9924541

[35] Foster C, Heimann KM, Esten PL, Brice G, Porcari JP. Differences in perceptions of training by coaches and athletes. S Afr J Sports Med. 2001 Jun 1; 8(2):3–7.

[36] Cross R, Siegler J, Marshall P, Lovell R. Scheduling of training and recovery during the in-season weekly micro-cycle: Insights from team sport practitioners. Eur J Sport Sci. 2019 Nov; 19(10):1287–96. Epub 2019/03/30. doi: 10.1080/17461391.2019.1595740 . 30922202

[37] Austin D, Gabbett T, Jenkins D. The physical demands of Super 14 rugby union. J Sci Med Sport. 2011 Feb; 14(3):259–63. doi: 10.1016/j.jsams.2011.01.003 21324741

[38] Cahill N, Lamb K, Worsfold P, Headey R, Murray S. The movement characteristics of English Premiership rugby union players. J Sports Sci. 2013 Nov 266; 31(3):229–37. Epub 2012/09/27. doi: 10.1080/02640414.2012.727456 . 23009129

[39] Tee JC, Lambert MI, Coopoo Y. GPS comparison of training activities and game demands of professional rugby union. Int J Sports Sci Coach. 2016 Sep; 11(2):200–11. doi: 10.1177/1747954116637153

[40] Gabbett TJ. Relationship between accelerometer load, collisions, and repeated high-intensity effort activity in rugby league layers. J Strength Cond Res. 2015 Jul; 29(12):3424–31. doi: 10.1519/JSC.0000000000001017 26196661

[41] Hulin BT, Gabbett TJ, Johnston RD, Jenkins DG. PlayerLoad Variables: Sensitive to Changes in Direction and Not Related to Collision Workloads in Rugby League Match Play. Int J Sports Physiol Perform. 2018 Feb 26; 13(9):1136–42. Epub 2018/03/16. doi: 10.1123/ijspp.2017-0557 . 29543076

[42] Dwyer DB, Gabbett TJ. Global positioning system data analysis: velocity ranges and a new definition of sprinting for field sport athletes. J Strength Cond Res. 2012 Mar; 26(3):818–24. doi: 10.1519/JSC.0b013e3182276555 22310509

[43] Barnard DV, Pote L, Christie C. Workloads of forward and backline adolescent rugby players: a pilot study. S Afr J Sports Med. 2020 May; 32(1):1–5. doi: 10.17159/2078-516X/2020/v32i1a7427 36818978 PMC9924503

[44] Quarrie KL, Raftery M, Blackie J, Cook CJ, Fuller CW, Gabbett TJ, et al. Managing player load in professional rugby union: a review of current knowledge and practices. Br J Sports Med. 2017 Aug 9; 51(5):421–7. Epub 2016/08/11. doi: 10.1136/bjsports-2016-096191 . 27506436

[45] Reinhardt L, Schulze S, Kurz E, Schwesig R. An Investigation into the Relationship Between Heart Rate Recovery in Small-Sided Games and Endurance Performance in Male, Semi-professional Soccer Players. 2020 Sep 10; 6(1):43. Epub 2020/09/11. doi: 10.1186/s40798-020-00273-8 . 32910327 PMC7483686

