# Supplementary material for: Position-specific workload of professional rugby union players during tactical periodization training
Source: PLoS One. 2024 Mar 29;19(3):e0288345. doi: 10.1371/journal.pone.0288345 (PMC10980212; doi:10.1371/journal.pone.0288345)
Supplement: S1 Data — (PDF) [file pone.0288345.s003.pdf]

editage

Editing Certificate

This document certifies that the manuscript listed below has been edited to ensure language and grammar accuracy and is error free in these aspects. The logical presentation of ideas and the structure of the paper were also checked during the editing process. Furthermore, a technical review of the manuscript contents was performed. The edit was performed by professional editors at Editage, a division of Cactus Communications. The author's core research ideas were not altered in any way during the editing process. The quality of the edit has been guaranteed, with the assumption that our suggested changes have been accepted and the text has not been further altered without the knowledge of our editors.

MANUSCRIPT TITLE

Position-specific workload of professional rugby union players during tactical periodization training

AUTHORS

Xiaopan Hu

ISSUED ON

February 13, 2023

JOB CODE

XIOPA_5

THE EDITAGE Promise QUALITY GUARANTEE

Vikas Narang

Vikas Narang
Chief Operating Officer - Editage

editage

Editage, a brand of Cactus Communications, offers professional English language editing and publication support services to authors engaged in over 1300 areas of research. Through its community of experienced editors, which includes doctors, engineers, published scientists, and researchers with peer review experience, Editage has successfully helped authors get published in internationally reputed journals. Authors who work with Editage are guaranteed excellent language quality and timely delivery.

GLOBAL :
+1(833) 979-0061 | request@editage.com

CHINA :
400-120-3020或021-6020-9400 | fabiao@editage.cn

CACTUS

IMPACT SCIENCE impact.science

researcher.life

CACTUS LIFE SCIENCES lifesciences.cactusglobal.com
