# Supplementary material for: Position-specific workload of professional rugby union players during tactical periodization training
Source: PLoS One. 2024 Mar 29;19(3):e0288345. doi: 10.1371/journal.pone.0288345 (PMC10980212; doi:10.1371/journal.pone.0288345)
Supplement: S2 Data — (PDF) [file pone.0288345.s004.pdf]

Request for Permission to Publish Content under CC-BY License

Dear Rights Holder or Representative,

I have submitted a paper for publication in a PLOS journal, and wish to include the content listed below in the paper. I'm hereby requesting your (or your company's or institution's) permissionto include the content in my paper. Please note that all PLOS journals are published under aCreative Commons Attribution License (CC BY), which allows for unrestricted use and distribution, even commercial, as long as attribution is given to the creator or rights holder of the content. See https://creativecommons.org/licenses/by/4.0/.

To grant me permission to use the content in my PLOS paper, please fill in the information below and then scan the completed form and send it to me at my email address.

Thank you.

My name:

Xiaopan Hu

My email address:

xiaopan.hu@ens-rennes.fr

Description of the content which I'm seeking permission to use (citation and/or title, and pasted screen shot, if applicable):

Table 1. Organization of the Pro D2 season and the arrangement of the two morphocycle models.
Figure 1. The two tactical periodization morphocycle models used for the practice session of a professional rugby union team.

Link to the Content:

N/A

* * *

On behalf of myself or the rights holder, I hereby grant the permission sought herein.

Signature of Party Granting Permission:

Date:

26.06.2023

Printed Name and Title:

Xiaopan Hu (PhD)
