# Supplementary material for: Position-specific workload of professional rugby union players during tactical periodization training
Source: PLoS One. 2024 Mar 29;19(3):e0288345. doi: 10.1371/journal.pone.0288345 (PMC10980212; doi:10.1371/journal.pone.0288345)
Supplement: S1 Text — (DOC) [file pone.0288345.s005.doc]

**Position-specific workload of professional rugby union players**

**during tactical periodization training**

Xiaopan Hu^1,2,3*^, Simon Boisbluche^4^, Kilian Philippe^3,5^, Olivier Maurelli^6^, Xiangyu Ren^1,2,3^, Shichang Li^1^, Bo Xu^1^, Jacques Prioux^1,2,3^

^1^ Sino-French Joint Research Center of Sport Science, College of Physical Education and Health, East China Normal University, Shanghai, China

^2^ Movement, Sport, and Health Sciences Laboratory, Rennes 2 University, Bruz, France

^3^ Department of Sport Sciences and Physical Education, École Normale Supérieure de Rennes, Bruz, France

^4^ Rugby Club Vannes, French Rugby Federation, Vannes, France

^5^ Movement, Balance, Performance, and Health Laboratory, University of Pau and Pays de l’Adour, Tarbes, France

^6^ Muscle Dynamics and Metabolism Laboratory, University of Montpellier, Montpellier, France

* Corresponding author:

Email: [xiaopan.hu@ens-rennes.fr](mailto:xiaopan.hu@ens-rennes.fr) (XPH)

**Author Contributions:**

**Conceptualization:** Simon Boisbluche, Jacques Prioux.

**Formal Analysis:** Xiaopan Hu, Xiangyu Ren.

**Investigation:** Xiaopan Hu, Xiangyu Ren.

**Methodology:** Simon Boisbluche, Xiaopan Hu.

**Project Administration:** Simon Boisbluche, Shichang Li, Bo Xu, Jacques Prioux.

**Supervision:** Shichang Li, Bo Xu, Jacques Prioux.

**Writing – Original Draft Preparation:** Xiaopan Hu.

**Writing – Review & Editing:** Xiaopan Hu, Kilian Philippe, Olivier Maurelli, Jacques Prioux.
